# Multiple Factors Causing Myopia and the Possible Treatments: A Mini Review

**DOI:** 10.3389/fpubh.2022.897600

**Published:** 2022-05-10

**Authors:** Ari Shinojima, Kazuno Negishi, Kazuo Tsubota, Toshihide Kurihara

**Affiliations:** ^1^Laboratory of Photobiology, Keio University School of Medicine, Tokyo, Japan; ^2^Department of Ophthalmology, Keio University School of Medicine, Tokyo, Japan; ^3^Tsubota Laboratory, Inc., Tokyo, Japan

**Keywords:** myopia, anthropometrics, Asian, axial length (AL), treatments

## Abstract

The myopia epidemic has become a global public health problem. Although myopia is progressing worldwide, the recent coronavirus infections 2019 (COVID-19) outbreak has spurred myopia progression. The current evidence-based treatments for humans are atropine eye drops, optical treatment with defocus, use of orthokeratology, extending proximity working distance, pausing from near work every half hour and increased time outside the home. Studies on myopia using animal models have been conducted for more than 40 years. In recent years, new mechanisms of myopia suppression have been revealed from animal experiments such as inflammation control, intraocular pressure control, light control, and the activity of early growth response protein 1 control. This mini-review provides a summary of the scientific evidence currently available on the control of myopia, and the possible treatments mitigating myopia.

## Graphical Abstract

Factors in myopia control and progression. 
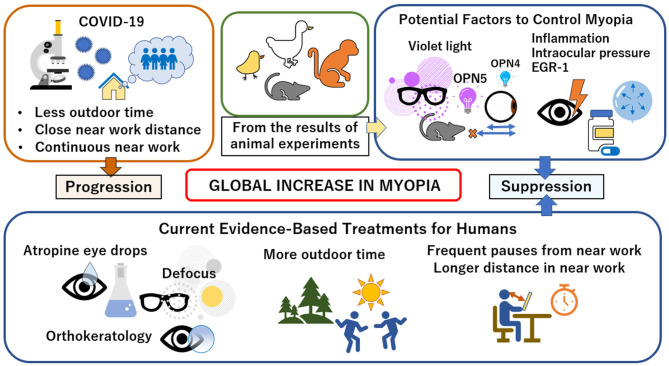

## Introduction

The widespread prevalence of myopia is a global public health problem. East Asia is experiencing unprecedented myopia. In China, between 10 and 20% of the population was nearsighted 60 years ago. Nowadays, in the teenage and twenties, maximum 90% are myopic (1). The myopia rate for 19-year-old males in Seoul, Korea is 96.5% (1). Myopia is increasing rapidly in other countries such as Europe and the United States, where about half of the young population is found to be myopic (1). We have reported that ~77% of schoolchildren between 6 and 11 and 95% of schoolchildren between 12 and 14 had myopia in two schools in Tokyo, Japan (2). Holden et al. projected that 50% of the global population will have myopia and the 10% will have high myopia by 2050, with the prevalence of myopia doubling (from 22% in 2000) and the prevalence of high myopia increasing 5-fold (from 2% in 2000) (3). Although myopia has been gradually increasing, studies on myopia using animal models have been conducted for more than 40 years. In 1977, Wiesel and Raviola became the first in the world to create an animal model of myopia after neonatal lid fusion in monkeys (4). Subsequently, from the fact that myopia did not progress in animals kept in the dark, they suggested that myopia is caused by changes in visual input and neurological mediation. Their experiments suggested that although the refractive state is largely genetically programmed, unusual visual experiences disturb the growth process of the eye after birth and trigger the development of axial myopia (5). Wallman et al. reported myopia models of chicks that are reared with white translucent eye occluders and are visually deprived of the nasal, temporal, or entire retina (6). As a result, myopia with a median of−15 diopters was limited to the portion of the retina that was deprived of vision (6). These studies have suggested that visual information can cause acquired myopia. It has been reported that near work can cause myopia even in humans (7). Huang et al. investigated 2-year refraction data every 6 months with a questionnaire; the protective behaviors of extending proximity working distance, pausing from near work every half hour and increased time outside the home from parents' self-reports on the prevalence and progression of myopia in 9–11 year olds in Taipei, Taiwan. The risk ratios were 0.71, 0.89, and 0.77 in each behavior. In a spherical equivalent analysis, each behavior also significantly reduced myopia progression from 6 to 24 months (7).

The novel coronavirus infections 2019 (COVID-19) has been affecting the world for 2 years as of March 2022. Natural experiments provided empirical evidence that Chinese schoolchildren who were studying at home during the COVID-19 epidemic were at higher risk of myopia progression (8).

It is not true that myopia progresses only when we are young and does not progress when we become adults. Lee et al. reported that in a cohort study of the general young population, they observed significant increases in myopia and axial length of 0.04 diopters and 0.02 mm per year, respectively, over the 8-year study duration. Among the 526 individuals who were free of myopia at baseline, the incidence of myopia between the ages of 20 and 28 years was 14% (9). In addition, females have a risk factor of high myopia (10, 11), and there may be gender differences in the development of myopia (12).

In this mini-review article, we searched the topic of myopia suppression treatment and experimental topics in the electronic databases PubMed from the inception until February 2022. The following keywords were used: “Myopia” alone or in combination with “choroid,” “hypoxia,” “genetics,” “inflammation,” and “light.” Relevant articles were also reviewed in this mini-review. This mini-review will provide a brief overview of the current scientific evidence on the control of myopia, and potential treatments to mitigate myopia.

## Choroidal Thickness and Myopia

Others, as well as our team, have reported that myopia is associated with subfoveal choroidal thickness which is significantly related to refractive error (13–15) and axial length (13, 16). Choroidal thickness is also reported to fluctuate in response to diurnal variation and changes in visual input (17–19).

The average subfoveal choroidal thickness in healthy humans ranges from 200 to 300 μm from age twenties to eighties (20). In chickens, the choroidal thickness of the central region is about 250 μm under normal visual conditions (17, 21). The choroid in chickens, increases in thickness by as much as 1 mm (over 17 diopters) to accommodate myopic defocus (a focused image in front of the retina). It compensates for many refractive errors by pushing the retina toward the image plane (21). There is the diurnal modulation of choroidal thickness in addition to the modulation of choroidal thickness due to refractive state, with a maximum thickness around midnight and a minimum thickness at noon, with a maximum width of 40 μm (17, 18). This diurnal rhythm in chickens is free-running under constant darkness, suggesting that it is driven by a circadian oscillator (22). Significant diurnal changes are known to occur even in healthy humans, and similar to the rhythm seen in chicken, the choroid has been found to be thicker at night and thinner during the day (19).

Pendrak et al. examined sources of chick choroidal extravascular fluid under conditions of two visually controlled ocular growths: goggle-induced myopia with an enhancement of ocular growth and delayed ocular growth in myopia recovery after goggle removal, and evaluated the evidence for changes in choroidal thickness. Fluorescein-dextran was injected intravenously as a tracer into 2-week-old chicks in each group of control, myopia and myopia recovery. As a result, suprachoroidal fluid protein concentration in myopic eyes at 1 h after intravenous injection fell significantly to 1.5% of plasma levels. On the other hand, recovery from myopia significantly increased the protein concentration of the suprachoroidal fluid to 30% of that of the plasma. They also reported that neither procedure affected the protein of suprachoroidal fluid in their contralateral eyes as controls (23). The altered levels of protein and marker dye in both myopic and recovering eyes suggest that choroidal circulation dynamics and capillary permeability are markedly altered (24).

## Hypoxia-Inducible Factor and Myopia

Thinning of the sclera has been observed in myopic animal models (25–27) and humans (28). Myopia has been thought to result from inadequate ocular axial extension and associated remodeling of the extracellular matrix resulting in reduced scleral strength and thickness.

Wu et al. reported that hypoxic exposure of 5% oxygen promotes myofibroblast transdifferentiation with down-regulation of type I collagen in human scleral fibroblasts and hypoxia-inducible factor-1α (HIF-1α) signaling promotes myopia *via* myofibroblast transdifferentiation. They also reported that 45 of the 145 myopia risk genes, about one-third, interact with genes involved in the HIF-1α signaling pathway (29).

Zhao et al. reported novel genome-wide association study (GWAS) gene set analysis revealed that the HIF-1α signaling pathway is significantly enriched in extremely myopic individuals with refractive error <-10 D. In addition, they clarified that downregulation of HIF-1α in the sclera caused hyperopia and upregulation caused myopia in mice. They speculated that myopia risk factor such as near work, may cause hypoxia of the sclera by severely reducing blood circulation in the choroid in humans (30). We will discuss this again in the Discussion section.

## Inflammation and Myopia

Inflammation in the etiology of myopia has not yet been fully assessed. However, there have been several reports on inflammation and myopia. Epidemiological observations have shown that allergic conjunctivitis children are at high risk in myopia (31). In an animal model, Wei et al. showed that ocular surface inflammation caused by mast cell degranulation alters corneal tight junctions, initiating the secretion of inflammatory cytokines in the cornea, which subsequently leads to retinal inflammation and promotes myopia progression (31). Lin et al. found from data on children in the National Health Insurance Surveillance Database under age 18 that the incidence of myopia was significantly higher in patients with type 1 diabetes and inflammatory diseases such as uveitis and systemic lupus erythematosus than in patients without inflammation (32).

Lin et al. also found that atropine downregulated inflammation in the Syrian hamster with an experimental myopia model, the monocular form-deprivation eye. They found that the expression of c-Fos, interleukin (IL)-6, nuclear factor κB (NFκB), and tumor necrosis factor (TNF)-α was increased in myopic eyes and decreased with atropine administration. They also found that the progression of myopia was slowed by cyclosporin A, but accelerated by lipopolysaccharide and peptidoglycan (32). Takahashi et al. reported that in Vogt-Koyanagi-Harada (VKH) disease, progression of myopia occurs with increasing axial length. In addition, the sunset glow fundus was more frequently observed in VKH patients with myopia progression than in patients without myopia progression, and the subfoveal choroidal thickness was found to be thinner (33). We investigated the inhibitory effect of lactoferrin on myopia onset and progression using a mouse model of lens-induced myopia. We found that oral administration of lactoferrin prevented the onset of lens-induced myopia in mice by modulating extracellular matrix remodeling through the IL-6-MMP-2 axis (34). Thus, these studies suggest that the suppression of inflammation may lead to the treatment of myopia.

## Light-Response and Myopia

Recently, melanopsin-expressing RGCs (mRGCs: melanopsin-expressing retinal ganglion cell) have been found to be widely involved in light-induced control of a variety of physiological functions in mammals, changing the way we analyze the non-image-forming effects of light.

Melanopsin (OPN4) is primarily expressed in mammalian retinal ganglion cells, which are essentially light-sensitive cells (ipRGCs) with sensitivity to the blue spectrum (35). Currently, six subtypes of murine mRGCs have been characterized based on light-response properties, dendritic arborizations, morphologies, and brain projections (36). To date, OPN4 has been thought to account for the majority of non-image-forming photoreception in the retina, but the discovery of OPN5, which is sensitive to UVA light at wavelengths from 315 to 400 nm, is adding complexity to the known ocular and non-ocular photoreceptor system (37).

Dysfunction in retinal melanopsin signaling alters refractive development in mice. Retinal dopamine signaling is reduced in form-deprived mice lacking melanopsin. Systemic L-3,4-dihydroxyphenylalanine (L-DOPA) treatment attenuates Form-deprivation myopia in melanopsin knockout mice. Melanopsin is vital for refractive development and slowing myopia progression (38). Thakur et al. reported that irradiating the human eye with red and green light causes axial elongation, while irradiating with blue light suppresses axial elongation (39).

We have shown that violet light (360–400 nm wavelength) suppresses axial elongation in an experimental chicken myopia model, and by expression microarray analysis, we found that the expression of the myopia suppressor gene early growth response protein 1 (*Egr-1*) is upregulated by violet irradiation. We also reported violet light can prevent experimentally induced myopia in mice. In addition, the effect depended on exposure time of day, and evening exposure was sufficient to prevent experimental myopia (40). Not only violet light, but blue light was also reported to suppress the effects of lens-induced hyperopic defocus, resulting in a significant decrease in axial length, while red and green light exposure resulted in a significant increase in axial length and thinning of the choroid, with or without defocusing (39).

In a retrospective clinical study, comparing two types of contact lenses (partial violet light-blocking and violet light-transmissive), the violet light-transmissive contact lenses reduced myopia progression at ages <20 (41). In addition, the violet light-transmissive phakic intraocular lens reduced myopia progression and axial length elongation compared with the non-violet light-transmissive type (42). We conducted a clinical study in children aged 6–12 years and found that the axial elongation supression rate in the violet light transmissive glasses group was ~20% over 2 years (43). Thus, these studies suggest the OPN5 pathway as a possible target for myopia treatment.

## Discussion

The increase in myopia worldwide is an important public health consideration. The mechanisms by which myopia occurs are not yet fully understood, and effective treatment options are limited. We discuss the possibilities and evidence for the treatment of myopia below.

The use of atropine in the treatment of myopia appears to vary from country to country (32). Lin et al. showed evidence that an inflammatory response is involved in myopia and their animal studies indicated that atropine and treatment with anti-inflammatory agents effectively inhibited the development of myopia (32). Atropine eye drops alone cannot completely suppress myopia, but they are clinically easy to prescribe. Therefore, it will be one of the treatment options.

Huang et al. reported the protective behaviors of extending proximity working distance, pausing from near work every half hour and increased time outside the home from parents' self-reports were found to have protective effects in diminishing myopia progression around 10 years-old children in Taipei (7). Therefore, home isolation and home study during COVID-19 may exacerbate worldwide burden of myopia (8). Indeed, Choi et al. reported that myopia progressed more rapidly in schoolchildren during periods of high lockdown procedures associated with COVID-19. Although, optical treatment with multiple segment-incorporated defocusing was significantly associated with slower myopia progression compared to monofocal lens treatment during locked-down periods (44). Nakamura et al. investigated the myopia suppression effect of orthokeratology in schoolchildren and found that orthokeratology treatment inhibited myopia progression by an average of 0.85 D over 2 years, regardless of orthokeratology lens design (45). In addition to medication, preventive lifestyle behaviors will help reduce the progression of myopia, and myopia suppression using devices such as optical treatment with defocus and orthokeratology will also be options for treatment.

Wu et al. suggested that HIF-1α signaling promoted myopia through myofibroblast transdifferentiation (29). This raises the question of whether oxygen administration can be a treatment option for hypoxic responses. Although different from the eye, there are many examples of oxygen administration in myocardial infarction. The clinical efficacy of routinely administered oxygen therapy in patients with suspected acute myocardial infarction without hypoxemia at baseline was previously uncertain. Hoffman et al. studied the DETO2X-AMI (the Determination of the Role of Oxygen in Suspected Acute Myocardial Infarction) trial, a routine oxygen replenishment therapy, in the treatment of patients with suspected myocardial infarction without baseline hypoxemia, and compared it to ambient air. The DETO2X-AMI verified that routine oxygen supplementation in patients with suspected myocardial infarction without hypoxemia did not reduce all-cause mortality at 1 year. In other words, in this report, systemic oxygenation did not make a decisive difference to room air, even though local hypoxia was suspected (46). From this report, it makes questionable whether simple administration of oxygen would be effective for sclera with suspected ischemia. Whereas, Kloner et al. studied the localized delivery of supersaturated oxygen therapy to myocardial infarctions. In their clinical trials, patients with anterior ST-segment elevation myocardial infarction, localized delivery of supersaturated oxygen therapy was shown to be safe and effective in reducing infarct size, improving cardiac function, and inhibiting adverse remodeling of the left ventricle (47).

Using swept-source optical coherence tomography angiography, a gradual decrease in choroidal vascularity with myopia severity has been reported (48). Decreased choriocapillaris blood flow was associated with thinner choroid and increasing myopia severity (48). This suggests that the oxygen supply to the sclera may possibly be reduced. From the evidence of localized oxygenation for myocardial infarction, localized oxygenation for the eye may possibly be a treatment option in the future.

The therapeutic approach for myopia suppression by controlling intraocular pressure has not yet been established. However, Liu et al. suggested that lowering intraocular pressure can inhibit scleral fibroblast activation, suppress scleral remodeling, and reduce scleral dilatation force, which slows down balloon-like eye dilation. They also suggested that intraocular pressure reduction would increase blood perfusion in the choroid, alleviate scleral hypoxia, and slow scleral remodeling (49). The hypothesis of myopia treatment using violet light is now beginning to yield results through animal and clinical experiments (40–42). We investigated natural agents that inhibit myopia based on *Egr-1* activity and found that crocetin, a dietary factor, may have protective effects against myopia progression (50). It has also been reported that in humans, reading black text on a white background makes the choroid 16 μm thinner in just 1 h, and reading white text on a black background makes the choroid about 10 μm thicker, suggesting that reading white text from a black screen or tablet may suppress myopia (51). Thus, research for myopia suppression, such as intraocular pressure control, violet light irradiation, crocetin intake, and contrast control, is becoming increasingly popular.

In conclusion, there may not be a single treatment solution from many research results. Further research and treatment to control myopia is expected.

## Author Contributions

The topic was devised by AS under the conceptualization and supervision of TK. AS used PubMed for literature review. All authors have read and agreed to the published final version.

## Funding

This work was supported by Tsubota Laboratory, Inc. The funding body took part in interpretation of data.

## Conflict of Interest

Outside the submitted work, KT reports his position as CEO of Tsubota Laboratory, Inc., Tokyo, Japan, a company producing myopia-related devices. The remaining authors declare that the research was conducted in the absence of any commercial or financial relationships that could be construed as a potential conflict of interest.

## Publisher's Note

All claims expressed in this article are solely those of the authors and do not necessarily represent those of their affiliated organizations, or those of the publisher, the editors and the reviewers. Any product that may be evaluated in this article, or claim that may be made by its manufacturer, is not guaranteed or endorsed by the publisher.
